# Role of Nanotechnology in Overcoming the Multidrug Resistance in Cancer Therapy: A Review

**DOI:** 10.3390/molecules27196608

**Published:** 2022-10-05

**Authors:** Suhail Ahmad Mir, Laraibah Hamid, Ghulam Nabi Bader, Ambreen Shoaib, Mohamed Rahamathulla, Mohammad Y. Alshahrani, Prawez Alam, Faiyaz Shakeel

**Affiliations:** 1 Department of Pharmaceutical Sciences, University of Kashmir, Hazratbal, Srinagar 190006, India; 2 Department of Zoology, University of Kashmir, Hazratbal, Srinagar 190006, India; 3 Department of Pharmacy Practice, College of Pharmacy, Jazan University, Jazan 45142, Saudi Arabia; 4 Department of Pharmaceutics, College of Pharmacy, King Khalid University, Abha 61421, Saudi Arabia; 5 Department of Clinical Laboratory Sciences, College of Applied Medical Sciences, King Khalid University, Abha 61421, Saudi Arabia; 6 Department of Pharmacognosy, College of Pharmacy, Prince Sattam Bin Abdulaziz University, Al-Kharj 11942, Saudi Arabia; 7 Department of Pharmaceutics, College of Pharmacy, King Saud University, Riyadh 11451, Saudi Arabia

**Keywords:** cancer, chemotherapy, multidrug resistance, nanotechnology, nanomedicine

## Abstract

Cancer is one of the leading causes of morbidity and mortality around the globe and is likely to become the major cause of global death in the coming years. As per World Health Organization (WHO) report, every year there are over 10 and 9 million new cases and deaths from this disease. Chemotherapy, radiotherapy, and surgery are the three basic approaches to treating cancer. These approaches are aiming at eradicating all cancer cells with minimum off-target effects on other cell types. Most drugs have serious adverse effects due to the lack of target selectivity. On the other hand, resistance to already available drugs has emerged as a major obstacle in cancer chemotherapy, allowing cancer to proliferate irrespective of the chemotherapeutic agent. Consequently, it leads to multidrug resistance (MDR), a growing concern in the scientific community. To overcome this problem, in recent years, nanotechnology-based drug therapies have been explored and have shown great promise in overcoming resistance, with most nano-based drugs being explored at the clinical level. Through this review, we try to explain various mechanisms involved in multidrug resistance in cancer and the role nanotechnology has played in overcoming or reversing this resistance.

## 1. Introduction

Cancer is a global burden, and as per the latest GLOBOCAN 2020, over 19.3 and 10 million new cases and deaths occurred in 2020, respectively; female breast cancer has surpassed lung cancer and is now the most commonly diagnosed cancer (11.7%), followed by lung cancer (11.4%), colorectal cancer (10%), and prostate cancer (7.3%) [1]. In mortality, lung cancer remains at the top [1]. As per World Health Organization (WHO) statistics 2019, in 112 out of 183 countries in the world, people die of cancer before attaining the age of 70 years [2]. Despite the world having advanced in science and technology, chemotherapy remains a promising option to treat cancer [3]. Conventional chemotherapy has greatly improved the decline in the mortality rate of several dreadful cancers, but its major problem is the killing of cancerous and noncancerous cells causing serious off-target effects such as hair loss, bone marrow depression, and other toxic effects [4]. Therefore, a significant percentage of cancer-related research over the past few decades has focused on creating medications that more precisely target tumor cells rather than normal cells [5]. Precision therapy has greatly advanced thanks to the development of targeted therapy, but there are still numerous unavoidable side effects, and drug resistance has long been an issue [6].

Over 90% of failures in chemotherapy are due to the development of resistance to the already available drugs; this resistance resembles infectious disease treatment resistance and is the most challenging aspect of treating and preventing cancers [7]. This has emerged as a major obstacle and allows cancer to proliferate in presence of a chemotherapeutic agent [8]. Significant resistance develops generally to repeated treatment with one kind of anticancer agent and then develops further towards similar or completely different drugs having a similar mechanism of action. This mechanism, known as multidrug resistance (MDR), can be intrinsic or acquired [9].

To overcome this problem, in recent years, nanotechnology-based drug dosage forms have been explored, which have shown great promise [10]. Most of these nanomedicines are heading toward clinical trials [11]. Nanotechnology has been used in medicine more and more over the past few decades, including applications for safer and more efficient tumor targeting, detection, and treatment [12,13,14,15,16,17,18,19,20]. Drug delivery methods based on nanoparticles (NPs) have demonstrated a number of benefits in the treatment of cancer, including good pharmacokinetics, accurate targeting of tumor cells, a decrease in adverse effects, and reduced drug resistance [21,22,23,24,25]. Nevertheless, nanomedicine-based formulations have some demerits, such as difficulty in physical handling due to smaller size, particle aggregation, limited drug loading, and burst release [19,20]. This review outlines the different mechanisms of cancer chemotherapy resistance and describes the different mechanisms such as the use of nanotechnology in overcoming multiple drug resistance.

## 2. Cancer Chemotherapy Resistance and Mechanism

**Figure 1:**
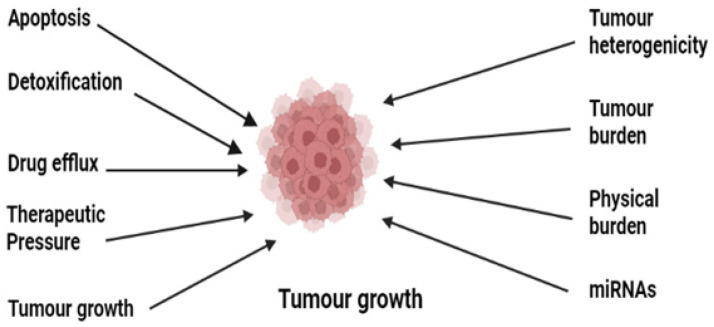
Illustration of the various possible underlying mechanisms in the development of drug resistance in cancer.

Cancer chemoresistance is a growing concern in medical oncology. Some cancers, including Hodgkin’s lymphomas, acute promyelocytic leukemia, and chronic myeloid leukemia, have been successfully understood and treated despite their complex pathophysiology [26]. The development of anticancer agents against these complex cancers has been achieved by understanding the deep mechanisms, and various drugs have been developed [7]. These mainly include the stimulation of immune response using interferon-alpha (IF-α) and inhibition of oncogenes or oncoproteins [27,28,29,30,31,32,33,34,35]. Many of them are still in practice; however, resistance has developed to the majority of them, which has ultimately affected patient survival [36]. There are various reported resistance mechanisms associated with cancer chemotherapy such as drug efflux, detoxification, stem cells, epithelial-to-mesenchymal transition, inactivation of the drugs before reaching the target, multidrug resistance, inhibiting cell death (apoptosis suppression), augmenting gene amplification and DNA repair of oncogenes, and alteration in the metabolism of drugs. Figure 1 shows the illustration of different possible mechanisms of chemotherapy resistance [37]. Drug resistance in cancer is believed to be due to intrinsic and acquired resistance; however, most cancers in clinical settings have become resistant owing to combinations of these factors [38]. In this review, we will describe a few important drug resistance mechanisms in cancer and will then focus our view on the use of nanotechnology in overcoming drug resistance in cancer. 

### 2.1. Role of Drug Efflux Pumps in Cancer Drug Resistance

The human genome encodes 48 members of drug efflux proteins called ATP-binding cassettes which are further classified into seven subgroups (ABCA, ABCB ABCC, ABCD, ABCE, ABCF, and ABCG) [39]. These proteins have a significant role in the development of drug resistance [40]. These proteins expel the drug out of the cell, thereby reducing the therapeutic concentration of the drug inside the cell [41]. Enough evidence suggests the overexpression of these proteins, especially multiple drug resistance protein 1 (MDR 1) known as P-glycoprotein, multiple drug resistance-associated protein (MDRA), and breast cancer resistant protein (BCRP), on cellular surfaces [42]. Normally these transporters help in pumping out toxins and foreign substances [43]. These transporters in general and P-pg in particular transport a range of substances including anticancer agents out of the cell, causing depletion of therapeutic concentration. Overexpression of P-pg in patients causes efflux of paclitaxel and doxorubicin and leads to resistance to these drugs [44]. This is evidenced by a study conducted on a genetically engineered mouse model (GEMM), where the tumor recurred due to upregulation of ABCB1a and b and was found to be cross-resistant to docetaxel also [39,45]. Another study shows the non-responsiveness of the tumor to olaparib, a PARP inhibitor, due to the overexpression of ABC1 a/b [46,47], thus confirming the overexpression of these efflux proteins in drug-resistant cancers.

### 2.2. Suppression of Apoptosis

Although apoptosis and autophagy are altogether different, they ultimately contribute to cell death [48]. Two different mechanisms in apoptosis contribute to cell death: (a) intrinsic, which involves the mitochondrial-mediated bcl2 proteins, Akt, and caspase-9, and (b) extrinsic, which involves death receptors on the cellular surface [49]. Ample evidence supports the initiation of human cancer from cancer stem cells (CSCs), and it is believed that these apoptotic pathways become dysregulated and lead to cancer chemotherapy resistance and tumor recurrence [50]. High levels of antiapoptotic proteins which are considered the hallmarks of cancer have been seen in drug-resistant cancers [51]. The antiapoptotic protein family which includes Bcl-2, Mcl-1, and Bcl-x_L_ has been seen at raised levels compared to proapoptotic proteins Bax, Puma, Noxa, Bak, Bil, and Bid, causing an imbalance between the pro- and antiapoptotic proteins which ultimately leads to cancer drug resistance [52]. The formation of the mitochondrial apoptosis-induced channel (MAC), which is formed by binding of tBid with Bax and Bak through activated caspase-8, is hindered by the downregulation of proapoptotic and upregulation of antiapoptotic proteins, which leads to the formation of resistant cancers by inhibiting the release of cytochrome C, a key protein for electron transfer in mitochondria [53]. This overexpression of antiapoptotic proteins is responsible for drug resistance in multiple cancers [54]. Additionally, overexpression of Nf-kB, P53, and PI3/AKT cell death-related receptors is also involved in chemoresistance [55]. In addition, apoptosis evasion through aberrant autophagy is another factor in the development of multiple drug resistance [56].

### 2.3. Drug Inactivation

Before a drug reaches the gastrointestinal tract or systemic circulation, some drugs that are in prodrug form interact with certain proteins which partially degrade, modify, and form complexes with other endogenous substances, leading to the activation of a drug [57]. Certain cancers have developed resistance due to decreased activation of prodrugs to active drugs [58]; the most prominent example is the mutation and downregulation of phosphorylation events in the conversion of AraC into AraC-triphosphate which is used in the treatment of acute myelogenous leukemia [8,59]. Several drugs metabolizing enzymes such as uridine diphosphate-glucuronosyltransferase, the glutathione-S-transferase family, and cytochrome P450 are muted one way or another and ultimately lead to resistance to already available drugs [60]. The overactivity of cytochrome p450 has been reported to lead to its resistance in breast cancer [61]. Detoxification of drugs by overproduction of glutathione has led to the development of resistance to many platinum compounds and alkylating agents such as cisplatin and doxorubicin [62]. Thus, mutations in phase I and phase II reactions either reduce the activity of drugs by increasing their detoxification or lead to the development of drug resistance by inactivating certain drugs.

### 2.4. Role of miRNAs in Cancer Drug Resistance

miRNAs are processed from RNA hairpin structures, which regulate genes in cancer, especially in resistant ones [63]. They are involved in apoptosis, cellular proliferation, stress tolerance, the cell cycle, and immune response [64]. Around 30% of human genes are regulated by miRNAs and have a role in tumor development [63]. Some act as protumor genes, some act as suppressor genes, and some act as both [65]. Studies conducted by various researchers provide evidence of miRNAs being involved in cancer drug resistance by either enhancing tumor cancer genes or having involvement in genes that are related to apoptosis, cellular proliferation, and the cell cycle [66]. Due to their tissue specificity, one kind of microRNA could be targeted by multiple microRNAs; hence the same miRNA can either promote or inhibit resistance to chemotherapy [67,68]. In breast cancer, upregulation of miRNA-21 downregulates phosphatase tensin homolog (PTEN) and thereby decreases the susceptibility of doxorubicin to cancer cells, while overexpression of PTEN inhibits miRNA-21 and reduces the resistance of breast cancer cells to doxorubicin [69]. Table 1 reports some of the miRNAs that regulate cancer chemotherapeutic drug resistance [70,71,72,73,74,75,76,77,78,79,80].

### 2.5. Tumor Microenvironment (TME)

The TME leads to the development of resistance by providing an environment rich in the stroma, immune cells, and vasculature which helps in the development of resistance by several mechanisms such as hampering drug absorption, restricting immune clearing of cancer cells, and stimulating factors for cancer cell proliferation [81]. Lactic acid produced by intermediate glycolytic intermediates results in a change in pH in cancer cells; this change in pH gradient results in neutralization and protonation of certain anticancer agents such as doxorubicin, thereby preventing them from entering the target site [82,83]. Hypoxia is a key factor in cancer drug resistance; its transcription factor hypoxia-inducible factor (HIF) is expressed in many cancers [84], which is supported by numerous studies involving HIF inhibitors as chemosensitizing agents in cancer. The TME also helps tumors to become resistant by providing an environment for metabolic reprogramming, DNA repair, and the immune microenvironment [85].

## 3. Nanotechnology and Cancer

**Figure 2:**
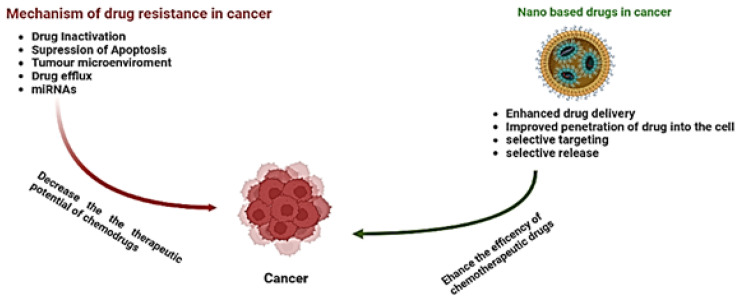
Illustration of the influence of nanotechnology on multidrug resistance in cancer.

**Figure 3:**
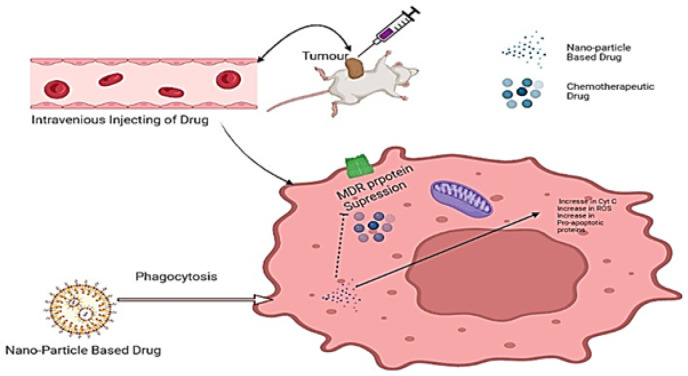
Illustration of the use of a nanoparticle-based drug delivery system in overcoming multidrug-resistant cancer.

Nanotechnology is an interdisciplinary field that has recently emerged as one of the most promising fields in the treatment of cancer [86]. Owing to this, an urgent need arises for the development of novel and innovative technologies and methodologies that could assist in characterizing tumors, recognizing micrometastasis and residuary tumor cells, and ascertaining whether or not a certain tumor has been removed completely [87]. The focus on nanotechnology for in vitro diagnostics and drug delivery has surged in recent years. This technology comprises only a few sections, albeit analytic ones, which are being assembled to triumph over the war against cancer [88]. Studies focusing on nano-based drugs have achieved tremendous achievements in reversing drug resistance either by active or passive mechanisms [89]. These nano-based drugs have successfully improved the efficacy of drugs, reduced off-target effects, and overcome drug resistance [90]. The advantage of nano-based drugs over conventional therapies is the selectivity of the target [91]. Over time, a number of nanoparticles have been designed and studied, such as metal nanoparticles, polymer-based nanoparticles, and nanovesicles such as liposomes and dendrimers which have greatly overcome chemoresistance in cancer [10]. Figure 2 and Figure 3 show the possible mechanisms of resistance and how nanotechnology helped in achieving efficient chemotherapy and overcoming resistance.

As already discussed, chemotherapy faces several unavoidable problems such as short half-life, cytotoxicity, lack of selective targeting, poor solubility, and multiple drug resistance. Nanomaterial-based chemotherapy, chemodynamic therapy (CDT), photodermal therapy (PDT), molecular therapy, sonodynamic therapy (SDT), photothermal therapy (PTT), and targeted therapy are currently being used in cancer treatment. In addition, an ample number of studies on certain therapies such as signal modification therapy, immunotherapy, nucleic-acid-based therapy, anti-angiogenesis therapy, therapies regulating apoptosis, and molecular therapy have been conducted in recent years [92]. Table 2 summarizes the different types of nanoparticles that have been explored in cancer research [93,94,95,96,97,98,99,100].

### 3.1. Targeting Mechanism of Nanoparticles in Chemotherapy

**Figure 4:**
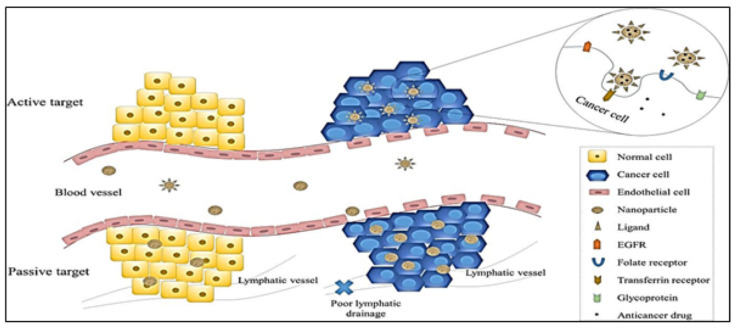
Schematic diagram of active and passive targeting of nanocarriers in cancer cells. Image reproduced with permission from reference [101].

Several studies have been performed to determine the mechanism through which nanoparticles execute their action against tumor cells. Before knowing the mechanism of action, it is crucial to know the interaction between cancer cells and nanoparticle-based drugs. Nanocarriers execute their mechanism of action in two ways, namely active targeting and passive targeting. Figure 4 illustrates the two types of targeting mechanisms of nanoparticles in cancer.

#### 3.1.1. Passive Targeting

Shreds of evidence support that the proliferation of tumor cells causes neovascularization and very large pores in vessels, leading to a decrease in the permeability of tumor cells compared to normal cells [102]. Passive targeting is achieved by the enhanced permeability and retention (EPR) (one of the driving forces for passive targeting) effect caused by retention of NPs due to poor lymphatic drainage associated with cancer cells, thus allowing nanocarriers to release the drug at the target site [103]. In addition, this EPR effect is achieved by the small particle size of NPs, which have better permeability [104,105] compared to larger particles such as conventional drugs which are likely expelled from the cell by the immune system [106].

#### 3.1.2. Active Targeting

Receptors or molecules such as siRNAs, proteins, vitamins, amino acids, monoclonal antibodies, and peptides that are expressed on the surfaces of cancer cells are utilized to achieve the active target mechanism of nanoparticles; in other words, this is achieved by the direct interaction between ligands and receptors. The ligand-mediated target of nanoparticles in cancer cells helps these particles in distinguishing between tumor cells and healthy cells [107,108]. This interaction leads to receptor-mediated endocytosis allowing NPs to release the drug at the target site [109,110]. The targeting ligands used for active targeting are usually monoclonal antibodies or antibody fragments or non-antibody ligands.

### 3.2. Polymeric Nanoparticles

Polymeric nanoparticles are defined as colloidal nanocarriers, having a submicron size of 10–1000 nm. Sustained release to the target site is achieved by these nanoparticles [111]. The dimensions of nanocarriers are very important for targeting cancer. The dimension of colloidal nanocarriers for reaching cancer cells should be less than 200 nm [112]. It is difficult for nanoparticles with larger dimensions to reach cancer cells. API is encapsulated on the surface of polymeric nanoparticles, forming a nanosphere and nanocapsule. Polyacrylamide, polystyrene, polymethyl methacrylate, and polyacrylate are some of the non-biodegradable nanomaterials that have been used to fabricate these nanoparticles [113,114]. As there were certain limitations with the use of these materials, such as toxicity and other pharmacokinetic problems, biodegradable polymers such as poly(lactic-co-glycolic acid), poly amino acid, and polylactic acid have been introduced to overcome these limitations. Table 3 lists some of the polymeric nanoformulations that have been recently investigated with a positive outcome [115,116,117,118,119,120].

### 3.3. Extracellular Vesicles

Extracellular vesicles are used in long-distance communications because they contain protein, RNA, and DNA [121]. They have lipids similar to the cell, thus enabling them to escape the immune surveillance of the body and interact with the target site easily [122]. There are several reports on the use of these extracellular vesicles in combatting multiple drug resistance in several cancers [98,123]. Hadla et al. and colleagues reported that exosomes loaded with doxorubicin showed better cytotoxicity and reduced accumulation of doxorubicin in the heart when compared with free doxorubicin [98]. Another study conducted by Jeong et al. and colleagues studied the use of exosomes to deliver mRNA-497 in a lung cancer cell line (A549) and found that tumor growth, as well as expression of associated genes, was suppressed, indicating this exosome-mediated miRNA therapeutic can be used in targeted cancer therapy to reduce cancer drug resistance [124]. Table 4 lists some of the EVs developed for overcoming MDR in cancer [125,126,127,128,129,130,131].

### 3.4. Using Nanocarriers in the Delivery of Pooled siRNAs in Combatting MDR in Cancer

In addition to the strategies mentioned above, pooling siRNAs using nanocarriers has emerged as a novel tool for overcoming multidrug resistance, which is confirmed by a study conducted by Chen and coworkers in 2015 [132,133]. This technique inhibits the no flux and efflux-related protein pumps which are involved or overexpressed in multidrug resistance, thereby enhancing the efficacy of already existing drugs. In order to improve the treatment result against resistant tumors, He et al. (2014) carried out a study on the loading of siRNA with cisplatin into nanoscale metal–organic frameworks (NMOFs) [134]. Bcl-2, P-gp, and survivin siRNAs were employed by He et al. to silence genes. In this study, cisplatin (prodrug-based bisphosphonate bridging ligand) was linked with Zn^2+^ metal and coated with a cationic lipid layer, which enabled the adsorption of pooled siRNA. This increased drug release increased the cellular uptake of pooled siRNA and cisplatin [135]. Table 5 lists some pooled siRNAs combined with gene therapy to overcome MDR in cancer [136,137,138,139,140,141,142,143,144].

### 3.5. Using Nanoparticle-Based Combination Therapies in Overcoming Multidrug Resistance in Cancer

Since conventional therapies have developed resistance to various classes of drugs by various mechanisms as described above, combination therapies using nanosystems that are sensitive to changes in pH, certain enzymes, and ROS in cancer cells have gained importance in cancer therapeutics [145]. Li and colleagues have reported that these systems efficiently release the drug into the tumor cell [146]. The tumor microenvironment acts as a physical and biological barrier to the drug as it controls and regulates its accumulation in and outside the cell, thereby helping in inducing drug resistance [147]. Table 6 summarizes the list of nanoparticle-based combinations as a strategic tool in overcoming drug resistance in cancer [144,148,149,150,151,152].

### 3.6. Application of Nanotechnology in Antibody-Mediated Targeting in Cancer

The immune response educed by tumor cells lacks adequate strength to counter the cellar response to external stimuli, and thus monoclonal antibodies have gained importance and have been used to counter tumor cells. Nanoparticles have been conjugated with antibodies that are specific against different tumor antigens [153]. These conjugated antibodies execute anticancer activity through various mechanisms, namely the inhibition of growth and the induction of apoptosis which is usually suppressed in resistant cancers [154]. These NP-mediated antibody targetings induce both complement and antibody-mediated cytotoxicity [155]. Researchers encapsulated the hepatic cancer therapeutic gene (HSV1tk) by preparing hollow protein nanoparticles. These nanoparticles were modified to carry the surface antigen of the hepatitis B virus so that its cells recognize it and form particles. This was tested in an animal model, and the results were satisfactory in delivering the gene into the target site after intravenous administration [155]. Another study conducted by Wartlick and coworkers designed and developed a biodegradable nanoparticle whose surface was altered by attaching a biotin-binding protein called NeutrAvidin; trastuzumab (HER2 receptor-specific antibody) was then conjugated to its surface for specific targeting of HER2-overexpressing cells, and this effect was confirmed by confocal laser scanning microscopy [156,157].

### 3.7. Application of Natural Polyphenol Nanotechnology in Reducing Multidrug Resistance in Cancer

Many studies showed that natural polyphenols have a great role in overcoming MDR in cancer. The commonly used polyphenols for this purpose are curcumin, resveratrol, and epigallocatechin-3-gallate (EGCG) [158,159]. Nanotechnology-based formulations of natural polyphenols attracted much attention due to several reasons. Several investigations have been carried out on the role of polyphenols in designing nanoformulations for drug targeting [158]. Gold nanoparticles based on gelatin–doxorubicin and EGCG were prepared and characterized for targeting prostate cancer. These gold nanoparticles coated with gelatin–doxorubicin and EGCG were found to increase the cellular uptake of doxorubicin [160]. Some studies also revealed that EGCG is able to reduce gold ions; this enabled the enhancement of gold nanoparticles, which showed higher drug uptake by tumor cells [161,162,163]. EGCG-based nanoformulations have also been found suitable for targeting drugs to non-small-cell lung carcinoma cells [164]. Curcumin-based nanoparticles have been found suitable for targeting drugs to brain and breast cancer [165,166]. Resveratrol nanoparticles have been shown to provide protection against UV light in various targets [167]. Liposomal-based formulations of resveratrol have been found effective in targeting drugs to brain tumors in several studies [167,168,169]. Resveratrol-based nanoformulations have also been found effective in the treatment of other tumors, such as lung cancer, colorectal cancer, glioma carcinoma, hepatocarcinoma, and breast cancer [170,171,172,173,174].

## 4. Conclusions

Resistance to chemotherapy is as similar to infectious disease drug resistance and is a complex phenomenon that is educed by the suppression of apoptosis-related proteins, enhancement of DNA repair, inactivation of drugs, overexpression of efflux proteins, and miRNAs and leads to the failure of already available drugs. Thus, overcoming this resistance is a hot topic in cancer research currently. Nanotechnology has gained importance in recent years in many diseases and has been applied to cancer therapy as well to overcome this multidrug resistance either through passive or active targeting mechanisms. The use of nanocarriers to pool siRNAs, nano-based drug combination with chemotherapy, and antibody-mediated target action have been explored in many cancers. When compared to conventional drugs, these nano-based drugs have successfully improved biocompatibility, target selectivity, pharmacokinetics, and stability, while also simultaneously helping in reducing systemic toxicity and overcoming the burden of multidrug resistance. In addition, nanocarriers are platforms for combination therapy, thereby helping to combine targeting agents with cytotoxic agents to achieve the reversal of drug resistance. Based on evidence-based literature, nanotechnology has the potential to revolutionize cancer chemotherapy through its robust mechanisms and target selectivity. Nevertheless, most of the nanotechnology-based formulations have been tested in animal models instead of human models. Therefore, more studies are required on human beings in order to reach the commercialization of these nanotechnology-based formulations.

## Figures and Tables

**Table 1:** List of some miRNAs that regulate cancer chemoresistance.

miRNA	Target	Cancer Type	Drug Target	Reference
miR-7	MDR1	SCLC	Anthracyclines	[70]
miR-21	PTEN	Breast	Trastuzumab	[71]
miR-20a	MAPK1	Colorectal	5-Fluorouracil	[72]
miR-103/107	P-gp	Gastric	Doxorubicin	[73]
miR-196a	MDR1/MRP1	NSCLC	Cisplatin	[74]
miR-17-5p	PHIPP2	MCL	Topotecan	[75]
microRNA-34a	SIRT1, Bcl-2	Prostate	Paclitaxel	[76]
miR-96	XIAP	Colorectal	5-Fluorouracil	[77]
miR-499a	UBE2V2	Cervical	5-Fluorouracil	[78]
miR-RNA-449	NOTCH1	Ovarian	Doxorubicin	[79]
miR-320c	SMARCC1	Pancreatic	Gemcitabine	[80]

**Abbreviations:** MDR1: multidrug resistance mutation 1; PTEN: phosphatase tensin homolog; MAPK1: mitogen-activated protein kinase 1; P-gp: P-glycoprotein; MRP1: multidrug resistance-associated protein 1; PHIPP2: phage phi-PP2; SIRT1: sirtuin 1; XIAP: X-linked inhibitor of apoptosis protein; Bcl-2: B-cell lymphoma-2; UBE2V2: ubiquitin conjugated enzyme E2V2; NOTCH1: human gene; SMARCC1: protein; SCLC: small cell lung carcinoma; NSCLC: non-small-cell lung carcinoma; MCL: mantle cell lymphoma.

**Table 2:** Type of nanoparticles in cancer research.

Modification	Payload	Therapy Involved	Outcome	Reference
PLGA NP	PTX	Chemotherapy	There was improved efficiency in drug delivery compared with free PTX	[93]
PEG, transferrin-modified NP	Nucleic acids	Nucleic-acid-based therapy	Transfected leukemia cells with K562 showed high efficiency compared to nontargeted particles	[94]
Trastuzumab-modified NP	Docetaxel	Targeted therapy, chemotherapy	There was an overall increase in cytotoxicity in HER2-positive BT474 cells with no or minimal effect in but not in HER2-negative MCF7 cells	[95]
Trastuzumab-modified NP	PTX	Targeted therapy, chemotherapy	There was much better efficacy in treatment with low cytotoxicity to human breast epithelial cells	[96]
PLGA NP	Alantolactone Erlotinib	Targeted therapy	Significant induction of apoptosis was seen in cancer treated with NP-loaded drug	[97]
Exosome	Doxorubicin	Chemotherapy	Accumulation of the drug in heart of mice was reduced and an increase in cytotoxicity of doxorubicin was seen	[98]
Gold NP-encapsulated IONPs/Ag cores	ONPs/Ag	PTT	Gold NP complex acted	[99]
Trithiol-terminated poly-meth-acrylic acid-modified nanorods	Fe_2_P	SDT, PTT	It showed photodermal and therapeutic potential	[100]

**Abbreviations:** PLGA: poly(lactic-co-glycolic) acid; NP: nanoparticle; PEG: polyethylene glycol; IONPs: iron oxide nanoparticles; Ag: silver; PTX: paclitaxel; ONPs: organic nanoparticles; Fe_2_P: iron phosphide; PTT: photothermal therapy; SDT: sonodynamic therapy.

**Table 3:** Some of the polymeric nanoparticle formulations that have been recently explored.

Type	Drug	Targeting Agent	Name of Polymer Used	Result	Reference
Polymeric nanoparticle	Cisplatin	Cytokeratin-specific monoclonal antibody	Poly(d,l-lactide-co-glycolide) and polyethene glycol	Prevent metastasis	[115]
Polymeric nanoparticle	Paclitaxel	Monoclonal antibodies (antiHERT2)	Poly(d,l-lactic acid)	Selective targeting	[116]
Polymeric nanoparticle	Paclitaxel	Folic acid	Polylactic acid and polyethylene glycol	Enhanced drug accumulation in tumor	[117]
Polymer micelle	Doxorubicin	Folic acid	PEG-co-poly(lactic-co-glycolic acid)	Increased cellular uptake and cytotoxicity	[118]
Polymer micelle	Doxorubicin	Folic acid	PEG-poly(aspartate hydrazine doxorubicin)	Increased endocytotic cellular uptake	[118]
Polymeric nanoparticle	Doxorubicin	Cyclo-(1,12)-penITDGEATDGC (cLABL)	PGLA Poly d,l-lactic-co-glycolic acid	It showed enhanced cellular uptake	[119]
Polymeric nanoparticle	Mitomycin	Folic acid	mPEG poly(ethylene glycol) methyl ether	Targeted cellular uptake and enhanced tumor tissue distribution of the drug were achieved	[120]

**Table 4:** Some of the extracellular vesicles used in chemotherapy.

Nanocarrier	Drug/System	Cancer Type	Results	Reference
Acryl acid polyethylene glycol-modified exosome	Paclitaxel	Lung cancer	High loading capacity, better accumulation of cancer cells, and improved therapeutic outcome are the advantages	[125]
Exosome	Doxorubicin	Osteosarcoma	The anticancer effect was increased while cytotoxicity was reduced in myocardial cells when compared to free doxorubicin	[126]
Exosome	miR-497	Lung cancer	Suppression of tumor growth as well as a decrease in expression of genes associated with tumors	[127]
Microvesicle	Therapeutic mRNA/protein	Schwannoma	Microvesicles loaded with miRNA led to the conversion of the prodrug into active form and resulted in cell death	[128]
Extracellular vesicle	miR-101	Osteosarcoma	Inhibition and suppression of migration and cell invasion after administration of miR-101-loaded extracellular vesicles	[129]
Exosome–liposome hybrid NP	CRISPR/Cas9 system	Osteosarcoma	These hybrid nanoparticles can deliver the CRISPR/Cas9 system and have the potential to be used for cancer therapy	[130]
Exosome	Interferon-γ fusion protein	Prostate cancer	Induction of immune response against prostate cancer-derived exosomes and inhibition of tumor growth by exosomal vaccines	[131]

**Table 5:** Summary of some of the nanocarriers that can be combined with gene therapy and chemotherapy for overcoming multidrug resistance in cancer.

Target	Gene	Nanocarrier	Chemoagent	Drug-Resistant Cell Line	Reference
P-gp	siRNA	PDA-coated mesenchymal stem cell (MSC)	Doxorubicin	MCF-7/ADR	[136]
		Chitosan nanoparticle	Doxorubicin	HepG2/ADR	[137]
		Polymeric NP	Doxorubicin	MCF-7/ADR	[138]
	mRNA	Molecular beacon-based micelle	Doxorubicin	OVCAR-8/ADR	[139]
Survivin	siRNA	Hyaluronic acid NP	Cisplatin	A549/DDP	[140]
Bcl-2	siRNA	Polymeric NP	Doxorubicin	HepG2/ADR MCF-7/ADR	[141]
GAPDH	siRNA	Liposome	Paclitaxel	HeLa, MCF-7	[142]
Autophagy	siRNA	Polymeric NP	Doxorubicin	A549/ADR	[143]
P-gp, Bcl-2, survivin	siRNA	Coordination polymerMOF	Cisplatin	SKOV-3	[144]

**Table 6:** Some of the latest nano-based drug combinations to overcome MDR in cancer.

Drug Delivery System	Treatment Strategy	Loaded with	Cancer Type	Reference
Nanoparticulate targeting mitochondria	Downregulation of pump-related proteins that are involved in drug resistance	Mitochondrial complex, P-gp siRNA	Breast cancer	[144]
Nanoparticle–peptide drug biconjugate	Enhancement of efficient drug delivery and release	Doxorubicin peptides	H69AR	[148]
Folate-decorated polymersome	Combining chemotherapy with P-gp inhibitors	Paclitaxel, doxorubicin, and tariquidar	MDR breast cancer	[149]
Polymer–drug conjugate	Bypassing of pumps related to drug efflux	Doxorubicin	Breast cancer	[150]
Zinc oxide nanoparticle	Synergistic autophagy with increased reactive oxygen species generation	Doxorubicin and zinc oxide	MCF-7	[151]
Liposome	Its controlled drug release promotes drug accumulation in cancers	Docetaxel (DTX) and dexamethasone (DEX)	KBv Human epidermoid carcinoma	[152]

## Data Availability

This study did not report any data.
